# Gastric Mucosa Pathology in Rats with Precancerous Lesions of Gastric Cancer with Spleen Deficiency and Blood Stasis

**DOI:** 10.1155/2022/1366597

**Published:** 2022-09-24

**Authors:** Qiu-yue Li, Peng-hui Yang, Xin Huang, Xin-long Li, Min-yi Luo, Bi-juan Xiao, Zi-ming Zhao, Si-yi Li, Hua-feng Pan

**Affiliations:** ^1^Guangzhou University of Chinese Medicine, Guangzhou 510405, China; ^2^Guangdong Provincial Second Hospital of Traditional Chinese Medicine (Guangdong Provincial Engineering Technology Research Institute of Traditional Chinese Medicine), Guangzhou 510095, China; ^3^Joint Laboratory for Translational Cancer Research of Chinese Medicine of the Ministry of Education of the People's Republic of China, Guangzhou 510405, China; ^4^International Institute for Translational Chinese Medicine, Guangzhou University of Chinese Medicine, Guangzhou 510405, China; ^5^Dongguan Institute of Guangzhou University of Chinese Medicine, Dongguan 523808, China

## Abstract

**Objective:**

This research aimed at better understanding the histopathological development of precancerous lesions of gastric cancer (PLGC) and organelle ultrastructure changes.

**Methods:**

Sprague-Dawley rats were randomly assigned to the model and control groups. Model rats drank N-methyl-N′-nitro-N-nitrosoguanidine solution, while control rats drank pure water ad libitum. At 1, 3, 5, 6, and 8 months after the start of feeding, eight rats were randomly chosen from each group, and gastric mucosa tissues were removed for histopathological analysis. H&E staining was applied to analyze the pathological histological structure of the rat gastric mucosa via a light microscope, and the ultrastructural changes were observed via a transmission electron microscope.

**Results:**

Gastric mucosal pathologies of model rats such as mucosal atrophy, intestinal metaplasia, inflammatory lesions, and even intraepithelial neoplasia deteriorated over time. The endoplasmic reticulum gap widened, the mitochondrial endothelial cristae were disrupted, the nuclear membrane thickened, and chromatin condensed with heterotypic alterations in the main and parietal cells. Additionally, endothelial cell enlargement and thickening of the microvascular intima were seen.

**Conclusion:**

Our research showed that the PLGC progression of rats is correlated with the pathological alteration axis of “normal gastric mucosa-gastric mucosa inflammatory changes-intestinal metaplasia with mild dysplasia-moderate to severe dysplasia.” Ultrastructure analysis of model rats is compatible with the structural changes in the gastric mucosa with spleen deficiency and blood stasis. The pathological evolutionary axis and ultrastructural analysis are helpful for evaluating potential novel herbal therapies for PLGC.

## 1. Introduction

Gastric cancer (GC) is one of the most common cancers worldwide, with the fifth highest morbidity rate and the fourth highest mortality rate among malignant tumors [1]. Despite significant medical progress, the five-year survival rate of patients is still less than 30% due to the high risk of recurrence and metastasis [2]. According to the Correa hypothesis, GC goes through the progress of “normal gastric mucosa-chronic nonatrophic gastritis-chronic atrophic gastritis (CAG)-intestinal metaplasia (IM)-dysplasia (Dys)-gastric cancer” [3]. This sequence of events may last several years, and long-standing inflammation is the main pathogenic factor leading to GC progression [4].

Atrophic gastritis with IM and/or Dys is referred to as precancerous lesions of GC (PLGC) because of its high risk of cancer [5, 6]. Blocking or delaying PLGC progression has been proven to be an effective way to prevent GC [7]. Traditional Chinese medicine states that if the spleen is weak, food is not digested. Shui et al. demonstrated that the weakness of the spleen and stomach is the key factor to the development of PLGC [8]. Although *Helicobacter pylori* has been recognised as a major risk factor, rare biomarkers are predicting the progression of PLGC and development of GC [9]. Further research on the mechanism of PLGC generation and development is critical for the clinical treatment of GC. Therefore, it is essential to develop and explore these animal models. Currently, some modelling has been used to induce PLGC, including physical factor modelling, chemically induced modelling, biological modelling, immune intervention modelling, and multifactorial modelling [10]. N-Methyl-N′-nitro-N-nitrosoguanidine (MNNG) has been recognised as an ideal modelling agent of PLGC with spleen deficiency and blood stasis and has been widely used in many research [11, 12].

In this research, a MNNG-induced rat model was performed to explore the pathological state of PLGC. We hope to provide a theoretical basis for the search of novel herbal treatments for PLGC via the pathological evolutionary axis and ultrastructural analysis.

## 2. Methods

### 2.1. Experimental Animals

Healthy male Sprague-Dawley (SD) rats (4–6 weeks old, 180 ± 20 g, *n* = 80) were purchased from the Animal Experimental Centre of Guangzhou Chinese Medical University. The experimental animal production licence number was SCXK (Guangdong) 2018–0034, and the animal use licence number was SYXK (Guangdong) 2018–0001. All rats were raised at the Animal Experimental Centre of Guangzhou Chinese Medical University in a specific pathogen-free environment (20 ± 2°C; 50–70% humidity) with adequate food and water. This study was approved by the Experimental Ethics Committee of Guangzhou Chinese Medical University. The design satisfied the principles of safety and fairness. Experimental animals met the national requirements for medical experimental animals.

### 2.2. Reagents, Materials, and Instruments

N-Methyl-N′-nitro-N-nitrosoguanidine (MNNG) was purchased from Kasei Kogyo Co. Ltd. (Tokyo, Japan). A total (5 g) was prepared with distilled water at 2000 *μ*g/mL and stored in a light-proof bottle at 4°C. Absolute ethanol, methanol, xylene, hydrochloric acid, and neutral resin were purchased from Sinopharm Chemical Reagent Co. Ltd. Alcian blue staining solution and periodic acid-Schiff (PAS) staining kits were purchased from the Wuhan Google Biological Company. H&E staining kits were purchased from Nanjing Jiancheng Technology Co. The dehydrator, paraffin embedding machine, paraffin slicer, blade, and tissue spreader were purchased from Shanghai Leica Instruments Co., Ltd. Tissue pads, slides, and cover glass were purchased from Jiangsu Shitai Experimental Equipment Co., Ltd. An optical microscope was provided by Nikon (Japan).

### 2.3. Animal Model

During the first week of feeding, the model rats freely drank the MNNG solution at doses of 100, 120, 150, and 200 *μ*g/mL. The basic conditions of the rats were observed to minimise mortality. The model rats were free to drink 200 *μ*g/mL MNNG solution, and the control rats were free to drink pure water. During the period of free consumption of MNNG solution, eight rats were randomly selected from the model and control rats at 1, 3, 5, 6, and 8 months. The selected rats were euthanized with 1% pentobarbital sodium after the last administration, and stomach tissues were collected for further experiments.

### 2.4. Histopathological Observation

The gastric tissue was obtained from the boundary of the lesser curved sinus. Some tissues were fixed in 10% neutral formalin, and dehydrated in alcohol, and xylene. Then samples were sectioned in paraffin (4-*μ*m sections) and stained with hematoxylin and eosin (H&E). Pathological abnormalities were observed using a light microscope (Nikon, Japan) and photographed.

### 2.5. Ultrastructure Observation

Other gastric tissues were sliced into 1-mm^3^ pieces and fixed in 4% glutaraldehyde overnight at 4°C. The samples were then fixed with 1% osmium tetroxide for 2 h. After being dehydrated in graded ethanol solutions, samples were immersed in a mixture of acetone and epoxy resin. Subsequently, tissues were embedded in epoxy resin-filled capsules and heated at 70°C overnight, followed by being cut into 60–80-nm sections. After staining with uranyl acetate and lead citrate, the sections were observed under a transmission electron microscope (HT7700, Japan) to capture the ultrastructural lesions of gastric mucosal epithelial cells.

## 3. Results

### 3.1. Pathological Changes in Gastric Mucosal Atrophy

**Figure 1:**
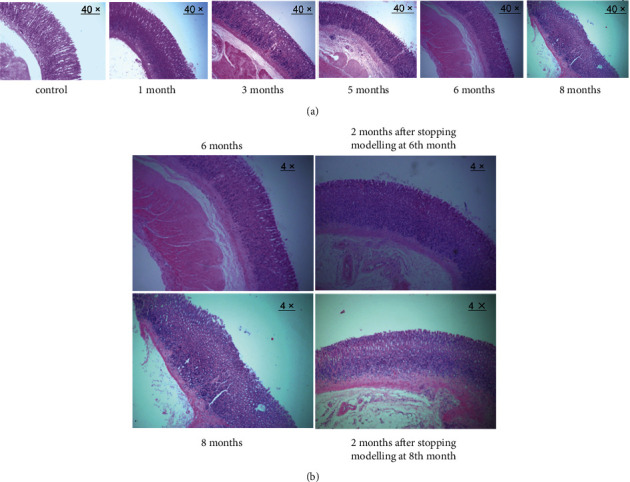
Pathological changes in gastric mucosal atrophy. (a) Pathological changes in rats at 1, 3, 5, 6, and 8 months of modelling (40 ×). (b) Pathological changes in rats after stopping the modelling at the 6th and 8th months (4 ×).

The gastric mucosa of the SD rats did not show obvious pathological (atrophy) changes at one month of modelling (Figure 1(a)). In comparison with control rats, the gastric mucosa of model rats gradually showed thickening of the mucosal muscle layer, a reduction in the number of small gastric pits, and an obvious decrease in glands at three months of modelling (Figure 1(a)). All of these changes suggest mild gastric mucosal atrophy. After five months of modelling, gastric mucosal atrophy gradually worsened to a moderate or severe grade (Figure 1(a)). No significant worsening of gastric mucosal atrophy was observed at 6–8 months after modelling (Figure 1(a)). The gastric mucosal muscle layer gradually thickened over modelling time at 3–5 months, but no longer thickened at 6–8 months, suggesting that gastric mucosal atrophy was no longer aggravated when it reached a certain degree.

To further investigate whether there was self-repair of gastric mucosal atrophy, the model rats were divided into a recovery group and a continued modelling group at the sixth or the eighth month of modelling. The rats in the recovery group were taken off the MNNG solution for two months to observe the automatic recovery of gastric mucosal histopathology. The H&E staining results showed that gastric mucosal atrophy in the recovery group did not improve significantly, suggesting that the pathological changes in gastric mucosal atrophy did not tend to self-repair without the intervention of external factors (Figure 1(b)).

## 4. Pathological Changes in IM

**Figure 2:**
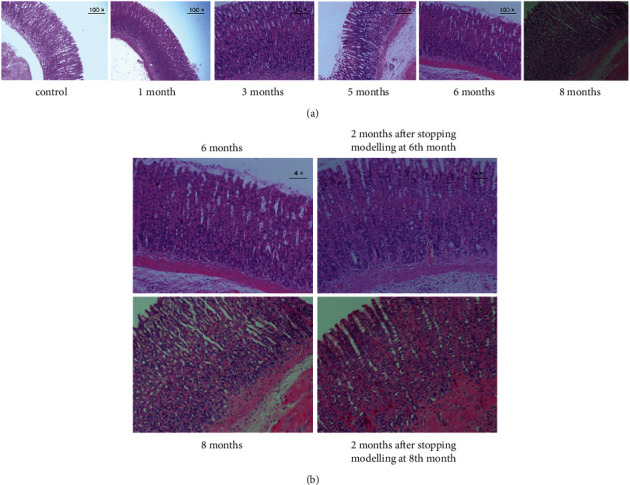
Pathological changes in IM. (a) Pathological changes in rats at 1,3, 5, 6, and 8 months of the modelling (100 ×). (b) Pathological changes in rats after stopping modelling at 6th and 8th months (4 ×).

After freely drinking MNNG solution for three months, the gastric mucosa epithelial cells in the model rats were partially replaced by intestinal-type epithelial cells compared with the control rats. Epithelial cells resembling small or large intestinal mucosa appear in the superficial layer of the gastric mucosa. IM was mainly formed in the upper and middle portions of the gastric mucosa, with a few vacuoles and mucus lakes (Figure 2(a)). After five months, the IM pathological changes were aggravated from the middle to upper superficial layers to the middle to lower and lower layers, and even throughout the whole gastric mucosa layer. With the aggravation of IM, U-shaped canals were observed. Rod-shaped heterotypic proliferative cells were observed in the gastric mucosa near the mucosal muscle layer with dark-stained and enlarged nuclei and an increased nuclear-cytoplasmic ratio (Figure 2(a)). At 6–8 months of modelling, the pathological changes in IM were not aggravated, suggesting that the gastric mucosa IM of rats was no longer aggravated when it reached the vicinity of the mucosal muscle layer (Figure 2(a)).

At sixth and eighth months of modelling, the model rats were divided into a recovery group and a continued modelling group to explore the self-recovery possibilities of IM. The rats in the recovery group were taken off the MNNG solution for two months. The IM in the recovered rats showed no significant improvement as revealed by H&E staining, implying that the pathological alterations in the IM did not tend to recover on their own without the intervention of external factors (Figure 2(b)).

### 4.1. Pathological Changes in Gastric Mucosa Dys

**Figure 3:**
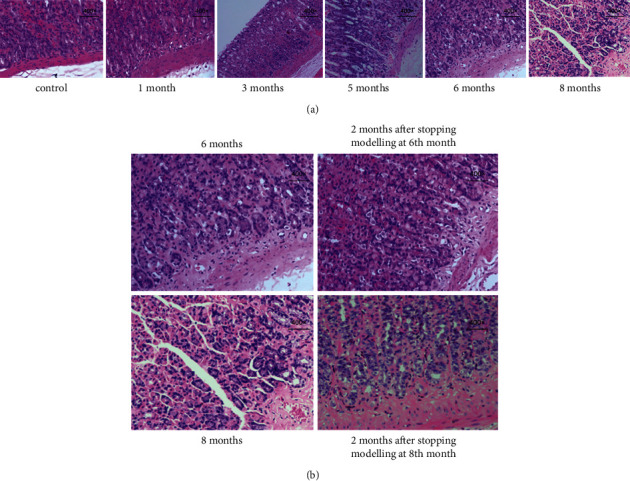
Pathological changes in gastric mucosa Dys. (a) Pathological changes in rats at 1, 3, 5, 6, and 8 months of modelling (400 ×). (b) Pathological changes in rats after stopping modelling at 6th and 8th months (400 ×).

At 1–3 months of modelling, the gastric mucosal epithelial cells in the model rats did not show aberrant differentiation of the glands and cells (Figure 3(a)). In gastric mucosal epithelial cells near the mucosal muscle layer, Dys that deviated from normal differentiation were observed after five months of modelling, including dark-stained and enlarged nuclei, increased nuclear-cytoplasmic ratio, and fusiform changes in nuclear morphology (Figure 3(a)). The cellular Dys became more severe after six months of modelling and began to infiltrate the mucosal muscle layer (Figure 3(a)). The Dys expanded from the basal layer to the middle and outer layers of the gastric mucosa at eight months in model rats with noticeable mucosal muscle layer infiltration, implying that the appearance of gastric mucosal Dys in rats occurs after IM and can worsen over time (Figure 3(a)).

At the sixth and eighth months of modelling, the model rats were separated into a recovery group and a continued modelling group to evaluate the feasibility of self-recovery of Dys. For the next two months, rats in the recovery group were not administered the MNNG solution. The gastric mucosa Dys was stagnant and tended to improve in the recovery rats that stopped modelling at the sixth month, while Dys continued to worsen in the recovered rats that stopped modelling at the eighth month. These results suggest that the recovered rats with mild Dys tended to improve, whereas those with severe Dys could not recover and improve by themselves (Figure 3(b)).

Under light microscopy, the model displayed considerable pathogenic alterations with a unique pattern. To investigate the impact of MNNG on the ultrastructure of rat gastric mucosa, an electron microscope was used to identify abnormal changes in the major organelles in the mucosa.

### 4.2. Ultrastructural Changes in Chief Cells of Gastric Mucosa

**Figure 4:**
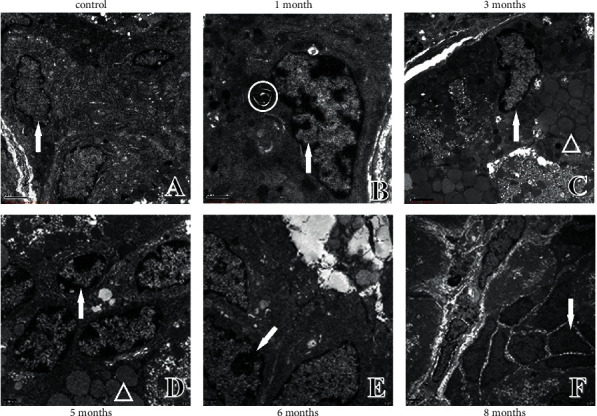
Ultrastructural changes in chief cells of the gastric mucosa. (a) Ultrastructure in control rats. (b) Ultrastructure in one-month modelling rats. (c) Ultrastructure in three-month modelling rats. (d) Ultrastructure in five-month modelling rats. (e) Ultrastructure in six-month modelling rats. (f) Ultrastructure in eight-month modelling rats. ↑: nucleus; ○: apoptotic body; △: pepsinogen granules.

The nuclear morphology and membrane thickness of the chief cells were normal, and the electron density was homogeneous in control rats (Figure 4(a)). In the model rats, pepsinogen granules with different electron densities and apoptotic bodies were observed; the nuclear membrane was thickened and chromosomes were condensed at one month of modelling (Figure 4(b)). At three months, the chief cells began to have a narrow nucleus, varying electron density of the cell bodies, and disappearance of some cristae (Figure 4(c)). At five months, vacuoles of varying sizes, large fused vacuolated pepsinogen granules, thickened nuclear membranes, and progressively more concentrated chromosomes were observed in the cells (Figure 4(d)). The nucleoplasm of the cells was aggregated, and the nuclear membrane thickened at six months (Figure 4(e)). As the modelling time continued, at eight months, the chief cell nucleus showed abnormal morphology and Dys without nonpolar arrangement, the cytoplasm was reduced, and the nuclear-cytoplasmic ratio increased and dissociated in cells without connection (Figure 4(f)).

### 4.3. Ultrastructural Changes in Parietal Cells of Gastric Mucosa

**Figure 5:**
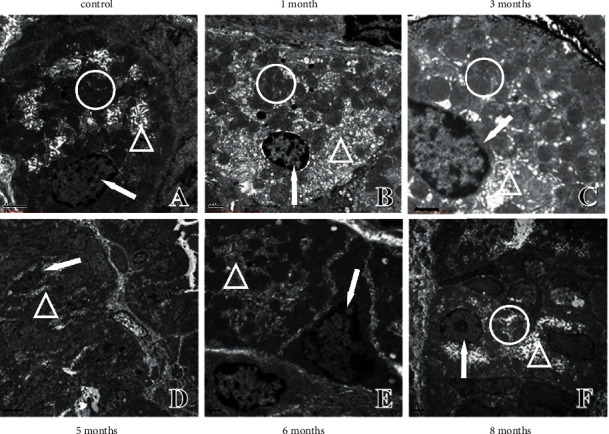
Ultrastructural changes in parietal cells of gastric mucosa. (a) Ultrastructure in control rats. (b) Ultrastructure in one-month modelling rats. (c) Ultrastructure in three-month modelling rats. (d) Ultrastructure in five-month modelling rats. (e) Ultrastructure in six-month modelling rats. (f) Ultrastructure in eight-month modelling rats. ↑: nucleus; ○: mitochondria; △: vacuolation; mucus lake; sinus tracts.

Parietal cells are acid-secreting cells whose main function is to secrete gastric acid. The ultrastructure of parietal cells in control rats under electron microscopy was as follows: normal nuclear morphology and membrane thickness, vacuolation and mucus lake distribution in the cytoplasm, and large mitochondrial electron density (Figure 5(a)). At one month of modelling, reduced electron density in mitochondria, mucilaginous intercellular secretory canaliculus, thickened nuclear membranes, and condensed chromosomes were observed in the rats (Figure 5(b)). At three months of modelling, the nucleus of parietal cells was further concentrated, mitochondrial cristae disappeared, and the intercellular secretory canaliculus was further mucilaginous (Figure 5(c)). Aggravation of vacuolation and bead-like vacuolar sinus tracts were observed at five months (Figure 5(d)). At six months, the number of intracellular sinus tracts increased, accompanied by a thickened nuclear membrane. The chromosomes were concentrated and showed conical morphology with individual heterotypic (Figure 5(e)). At eight months, in addition to being attached to the outer basement membrane of the glandular lumen, parietal cells with sinus tracts connected to the glandular lumen were primed to form free cells. Additionally, the nuclear membrane was thickened, chromosomes were condensed, and the number of anisotropic parietal cells was increased (Figure 5(f)). The increased number and abnormal morphological structure of the parietal cells over time suggested an association with decreased digestive capacity due to the affected gastric acid secretion function in PLGC.

### 4.4. Ultrastructural Changes in Endoplasmic Reticulum

**Figure 6:**
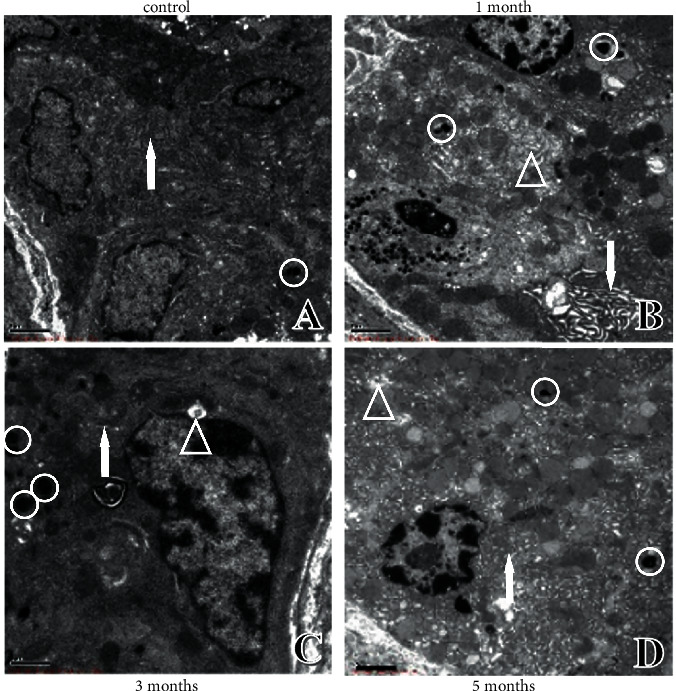
Ultrastructural changes in ER. (a) Ultrastructure in control rats. (b) Ultrastructure in one-month modelling rats. (c) Ultrastructure in three-month modelling rats. (d) Ultrastructure in five-month modelling rats. ↑: ER; ○: apoptotic body; △: vacuolation.

The endoplasmic reticulum (ER) is an interconnected lamellar vesicle and a small tubular lumen composed of biofilms, which are important organelles involved in intracellular protein synthesis, folding, lipid metabolism, and calcium storage. According to its morphology, the ER can be divided into rough and smooth ER. The rough ER has many ribosomes attached to the surface and is the site of protein synthesis and processing. The smooth ER has no ribosomes attached and functions in detoxification, lipid and glycogen synthesis, and regulation of intracellular Ca^2+^ homeostasis [13]. According to the cellular ultrastructure, the ER gap gradually widened, and ER ribosome granules were lysed, vacuolated, and even degraded over time. These changes may be related to apoptosis caused by the autophagic response of cells in the stress state (Figure 6(a)–6(d)). It has been shown that autophagy can occur in certain stress states as a protective mechanism for the cells. Bernales et al. first found in yeast that sustained ER stress and the unfolded protein response can cause selective autophagy against the ER, forming ER-only autophagosomes, and named it ER autophagy [14].

#### 4.4.1. Ultrastructural Changes in Mitochondria

**Figure 7:**
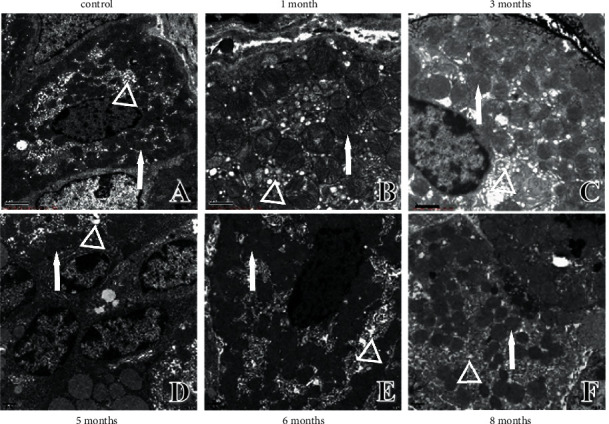
Ultrastructural changes in mitochondria. (a) Ultrastructure in control rats. (b) Ultrastructure in one-month modelling rats. (c) Ultrastructure in three-month modelling rats. (d) Ultrastructure in five-month modelling rats. (e) Ultrastructure in six-month modelling rats. (f) Ultrastructure in eight-month modelling rats. ↑: nucleus; △: vacuolation; mucus lake; sinus tracts.

Mitochondria provide energy for cellular life activities, and gastric acid secretion in parietal cells is mainly provided by the mitochondria [15]. The number of mitochondria in the cytoplasm was high and the structure was mostly normal, with a small number of alterations, such as widening of gaps and vacuolation in control rats (Figure 7(a)). With an increase in modelling time, the inner mitochondrial membrane cristae were broken and gradually disappeared, the colour of the mitochondrial matrix became lighter, the electron density decreased, and the number of autophagosomes increased in model rats (Figure 7(b) and 7(c)). In the middle and late stages of modelling, unclear mitochondrial membrane boundaries, membrane defects, and fusion were observed, and some mitochondrial membranes fused with the cell membrane (Figure 7(d) and 7(f)). Many studies have demonstrated that the main function of mitochondria is to regulate cell survival and apoptosis [16]. The reduced number and disruption of mitochondrial morphology can result in inadequate energy supply to the parietal cells, resulting in cell necrosis, decreased cell numbers, and decreased acid secretion. These results suggest that the altered mitochondrial morphology in this model may be related to decreased gastric acid secretion and abnormal apoptosis during PLGC.

### 4.5. Ultrastructure Changes in Gastric Mucosa Microvasculature

**Figure 8:**
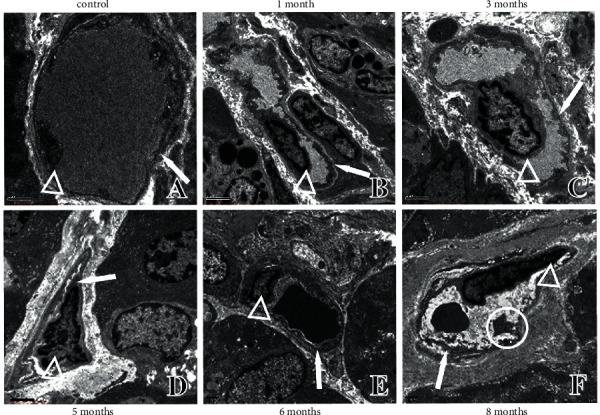
Ultrastructural changes in gastric mucosa microvasculature. (a) Ultrastructure in control rats. (b) Ultrastructure in one-month modelling rats. (c) Ultrastructure in three-month modelling rats. (d) Ultrastructure in five-month modelling rats. (e) Ultrastructure in six-month modelling rats. (f) Ultrastructure in eight-month modelling rats. ↑: microvasculature; ○: canal wall vegetation; △: endothelial cells.

The lumen of normal rat gastric mucosa microvasculature was large, the vessel wall was smooth and uniform in thickness, endothelial cells showed no heterogeneous changes (such as swelling and bulging), and there was no vascular occupancy (Figure 8(a)). The vessel wall thickness was not homogeneous in the early stage of modelling, the inner wall was not smooth, and there were no vascularly occupied endothelial cells (Figure 8(b) and 8(c)). In the middle stage, the endothelial cell lumen was elevated, the blood vessel lumen was occupied by endothelial cells, the endothelial cell nuclear membrane was thickened, chromatin was concentrated, and nuclear paging heterotypes appeared (Figure 8(d) and 8(e)). In the later stage, the nuclear membrane of swollen endothelial cells was thickened, the cytoplasm was convex toward the vascular lumen, the canal wall was uneven, and the basement membrane structure was unclear or even fractured and discontinuous. The pagination of the nucleus was heterogeneous, and the inner wall was not smooth, with large gaps and vegetation, suggesting increased permeability of the gastric mucosa microvasculature and a tendency of Dys (Figure 8(f)).

## 5. Discussion

PLGC, an important stage in the progression of GC, is IM and Dys of the gastric mucosa based on chronic atrophic gastritis [17]. PLGC changes from chronic nonatrophic gastritis to CAG, then to IM, Dys, and finally to GC, which is known as the Correa hypothesis [3, 18]. According to traditional Chinese medicine, spleen deficiency can lead to the dysfunction of the spleen and stomach, and the loss of transportation and transformation will lead to a lack of biochemical energy and blood. If the qi of the spleen is not elevated, there is no way to show spirit, thus resulting in various diseases, especially spleen and stomach diseases [19]. In Chinese medicine, PLGC belongs to “fullness,” “gastric pain,” and “noisy,” which are mostly caused by dietary and emotional disorders and are mostly seen in spleen deficiency. The spleen and stomach are the basis for people, and if the spleen and stomach are deficient for a long period of time, the qi is deficient to aid in blood flow, allowing the poisonous evil to invade, which gradually leads to phlegm, stasis, and poisonous symptoms [20].

In the present study, PLGC model rats with spleen deficiency were successfully constructed by freely feeding MNNG solution. Our findings revealed that model rats exhibited loss of appetite, depression, and loose stool, which can eventually deteriorate into intestinal-type GC over modelling time. All of these were similar to PLGC with spleen deficiency in terms of the clinical symptoms. The dynamic evolution of the model was illustrated by the results of the gastric mucosal pathology and electron microscopic ultrastructure. MNNG is currently recognised as a more desirable modelling agent that widely and effectively replicates CAG, PLGC, and GC models [21–23]. MNNG contains N-nitroso compounds, and nitrites are converted into nitroso compounds in the stomach, which in turn produce oxygen radicals. Oxygen radicals are cytotoxic and can damage the gastric mucosa, further accelerating the occurrence of PLGC and even leading to the development of GC.

In terms of pathological H&E staining, the rat gastric mucosa tissues were progressively aggravated along the pathological change axis of “gastric mucosal atrophy-IM-low-grade intraepithelial neoplasia-high-grade intraepithelial neoplasia,” which is in line with the Correa hypothesis. Our results showed that atrophic changes in the gastric mucosa and IM positively correlated with time. When the progression reached a certain level, the tendency of aggravation stopped, and the degree of lesions did not self-recover without intervention from external factors. Low-grade intraepithelial neoplasia in the gastric mucosa showed a positive correlation with time and could recover on its own, whereas high-grade intraepithelial neoplasia could not self-recover.

According to electron microscopy in the current study, both chief and parietal cells showed thickened nuclear membranes, dense chromatin, and heterogeneous nuclei in the late stage of modelling, which corresponded to the light microscopy results. Serious structural changes were observed in the ER, such as widening of gaps, increased autophagy, and vacuolisation, and even the formation of sinusoidal tracts, which are closely related to abnormal cell functions. These phenomena indicated that in the MNNG-induced PLGC rat model, cells gradually showed degeneration and even death, which critically affected cell function. Mitochondrial morphology was also damaged over the modelling time; for example, increased vacuolisation, decreased electron density, decreased or even disappeared endothelial cristae, and an increased number of autophagosomes. The microvasculature appeared swollen with endothelial cell degeneration occupying the official lumen of the microvasculature, with thinning and incomplete basement membranes, heterogeneous endothelial cell nuclear paging, and unsmooth walls with superfluous formation.

Liu et al. first proposed the theory of “spleen-mitochondria correlation” in 1983 [24]. The authors found that the number of mitochondria in parietal cells and the number of zymogen particles per unit area of chief cells were significantly reduced in patients with spleen and stomach deficiencies and gastroparesis. Compared with normal people and patients with liver and stomach disharmony, mitochondrial swelling, membrane defects, cristae fractures, and matrix pale changes can be seen in both parietal and plasma cells in patients with spleen and stomach deficiency and gastroparesis. It is believed that mitochondrial swelling and ER expansion may be caused by impaired mitochondrial function, lack of energy, impaired operation of the Na^+^-K^+^ pump, and excessive water accumulation in organelles. This is closely related to the inability of the spleen to transport water and dampness. There are various morphological signs of impaired protein synthesis in the rough ER, including damage, depolymerisation and degranulation of nucleus-proteasomes, and entry of water into the lumen of the ER, causing it to swell and expand. Several studies have found that splenic master water transport derangements are associated with changes in the expression and distribution of aquaporins and mRNAs [25–29]. These results suggest that the spleen is associated not only with mitochondria, but also with the rough ER. In addition, the gastric mucosa has a rich blood supply, which is an important material for the physiological and pathological evolution of the gastric mucosal and glandular epithelium. The pathological changes in endothelial cells, including swelling and occupation of the microvascular lumen, thinning and fracture of the basement membrane, heterogeneous nuclear paging of the endothelial cells, and the unsmooth wall with the formation of superfluous organisms, are related to impaired microcirculation in the gastric mucosa. These changes are consistent with the results of our previous study, indicating that spleen deficiency generates stasis over time, in line with the PLGC theory of “spleen deficiency, stasis, and toxin invasion” [30].

## 6. Conclusion

In the present study, the evolution of the PLGC progression was elucidated by histopathology and ultrastructure, which is consistent with the structural changes in the gastric mucosa in spleen deficiency and blood stasis, and has profound significance for the clinical diagnosis and prevention of PLGC. However, this study only observed the pathological changes in MNNG-induced PLGC model rats, and further studies on the treatment of PLGC with spleen deficiency and blood stasis and its mechanism are needed.

## Data Availability

The data used to support the findings of this study are included within the article, and they are available from the corresponding author upon request.
